# Reproducible Data Analysis With R in Laboratory Hematology

**DOI:** 10.1111/ijlh.70147

**Published:** 2026-05-15

**Authors:** Amrom E. Obstfeld

**Affiliations:** ^1^ Department of Pathology and Laboratory Medicine Children's Hospital of Philadelphia Philadelphia Pennsylvania USA; ^2^ Department of Pathology and Laboratory Medicine University of Pennsylvania Philadelphia Pennsylvania USA

**Keywords:** data analysis, laboratory hematology, quality assurance, R programming, reproducibility, spreadsheets

## Abstract

Laboratory hematology generates large, complex datasets that increasingly exceed the capabilities of traditional spreadsheet‐based analytical workflows. Despite their familiarity, spreadsheets obscure analytical logic, encourage manual data manipulation, and lack durable records of analytical steps, limiting transparency, auditability, and reproducibility. These limitations present specific challenges in clinical laboratory settings subject to regulatory oversight. Script‐based data analysis offers a practical alternative by encoding analytical workflows as explicit, ordered, and reusable operations. This review outlines the structural limitations of spreadsheet‐centered analysis, illustrates how these constraints contribute to analytical error, and introduces script‐based analysis using R as a reproducible framework suited to laboratory hematology. Practical applications are highlighted across method comparison, reference interval estimation, quality control, flow cytometry, and operational monitoring. The discussion also includes the importance of complementary technologies like integrated development environments, version control, and large language models. Adoption of reproducible, script‐based analytical tools such as R represents a foundational step toward improving data integrity, analytical rigor, and readiness for advanced analytical techniques in laboratory hematology.

## 1 Introduction

Laboratory hematology is a particularly data‐rich field within Pathology and Laboratory Medicine. Since the early cell counters of the 1950s [1], the discipline has seen a steady rise in both the volume and complexity of data, culminating in modern platforms that analyze thousands of multidimensional cellular events while generating clinical, research use only parameters, and detailed cell population data. The peripheral blood smear, long the foundational analytic method of the laboratory, historically resisted quantitative assessment because of the subjectivity of visual interpretation. Recent advances in digital microscopy and image analysis have begun to change this, extending the data revolution to morphologic review [2]. These innovations now generate large, information‐dense datasets, yet much of their potential remains unrealized. Embedded within routinely produced hematology data are biological signals that, if systematically analyzed, could substantially advance diagnostic practice and patient care [3].

A key approach to deriving meaningful insights from these increasingly complex datasets is through data analysis. Despite the rapid expansion in data size, dimensionality, and velocity, the analytical tools used by many laboratory professionals have changed little. Spreadsheet‐based workflows, most commonly implemented in tools such as Microsoft Excel and Google Sheets, remain common in routine clinical lab analysis. These platforms were originally designed for financial accounting rather than the demands of modern biomedical data analysis [4]. While such tools can support basic statistical and data analytic needs, they introduce major constraints, as discussed below.

There is a better alternative to spreadsheet‐centered process: script‐based data analysis workflows designed for statistical computing and reproducibility. Several mature environments exist for this purpose, including Python and R. This article focuses on R as a widely adopted platform within biomedical research and provides a practical introduction for the laboratory hematologist. It first outlines the risks inherent in spreadsheet‐driven workflows while contrasting them with an ideal reproducible process. It then introduces the framework of script‐based data analysis and demonstrates applied use cases spanning routine laboratory operations and clinical research. Finally, it provides guidance for getting started, including discussion of how large language models may lower the initial barrier to learning and using code‐based analytical tools.

## 2 The Case for a Paradigm Shift

Spreadsheets were originally designed to digitize physical “spread sheets,” ledgers used in financial accounting, and this lineage strongly shapes how they function today. This design heritage introduces major limitations when applied to biomedical data. First, spreadsheets encourage manual data manipulation which obscures analytical logic, limits transparency, and makes results difficult or impossible to reproduce [5, 6]. Second, spreadsheets impose a low ceiling on the types and scale of analyses that can be performed. Tasks such as multivariable modeling, robust regression, or analysis of high‐dimensional data from flow cytometry or digital imaging exceed what these platforms can reliably support.

### 2.1 Limits of Spreadsheet‐Based Analysis

Spreadsheets are designed with the expectation that users will manually interact with and modify data through direct manipulation. As an analysis becomes more complex, this design becomes fragile. Common operations such as reshaping, aggregating, or subsetting data are performed through manual rearrangement of cells, for example dragging columns, copying and pasting blocks of values, sorting rows in place, or extracting filtered or aggregated subsets into new sheets. The analytical process is fragmented into a series of manual actions rather than a continuous, inspectable pipeline.

At a structural level, spreadsheets are reactive, two‐dimensional grids in which data storage, analytical logic, and presentation are tightly coupled in the same space. When presented with the final result of an analysis, it is frequently unclear how that result was derived and even what portion of the spreadsheet is input data and what is the final output. While each individual change may be obvious at the time it is made, the order of these interactions is not preserved in a transparent way. Tracing the lineage of a result, how a given value was derived, filtered, aggregated, or transformed across successive steps, is challenging, so much so that it appears to be the inevitable effect of the spreadsheet to obscure this lineage [7].

**FIGURE 1:**
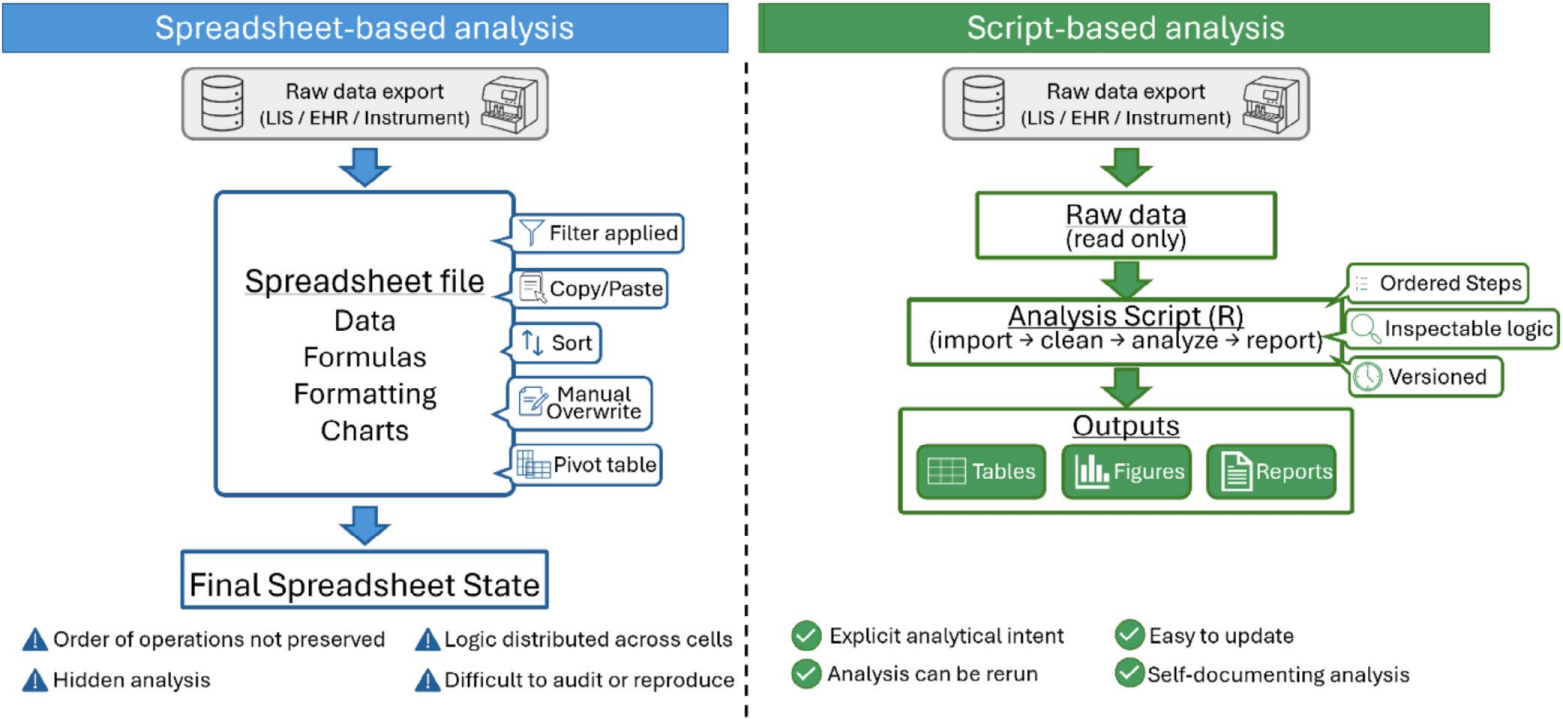
Conceptual comparison of spreadsheet‐based and script‐based analysis. Spreadsheet workflows tightly couple data, logic, and presentation and preserve only the final state of analysis, obscuring the sequence of analytical decisions. Script‐based workflows explicitly separate raw data, code, and outputs, preserving analytical intent in an ordered, reviewable, and reproducible form. In practice, script‐based workflows may operate on exported files or connect directly to databases and laboratory information systems, enabling automation without altering the underlying reproducibility model.

An illustrative example involving routine laboratory data can clarify this point. Consider a dataset of complete blood count orders filtered to a specific time frame, summarized by clinical unit and priority level (e.g., STAT vs. routine), and then used to perform row‐level operations based on those aggregated summaries, such as flagging elevated turnaround times relative to unit‐specific baselines. In a spreadsheet, this workflow requires filtering the dataset, generating a pivot table to compute aggregates, and then manually copying those summary values back into the row‐level table to support further row‐level calculations. Each step alters the state of the data, yet none of the transformations are captured as explicit, ordered operations. Because of the copy/paste action, small changes upstream, such as modifying a filter or adding new cases, do not automatically propagate, leaving downstream calculations silently inconsistent with the underlying data. By contrast, the same workflow can be expressed naturally as an explicit, ordered pipeline in a script‐based environment, where filtering, aggregation, and row‐level logic are encoded transparently and propagate consistently as the data change. Figure 1 presents a summary of the differences between working with spreadsheets and using structured pipelines.

### 2.2 Harmful Automation

As spreadsheet software was originally designed to streamline business and financial workflows, its automation and manual interface are optimized for the assumptions of those fields. The internal logic used to reduce friction, such as its automated correction logic and data type conversion, infers intent based on patterns found in those fields. In a scientific or clinical context, however, these same features can introduce subtle but consequential errors. Several noted examples illustrate how these “helpful” behaviors can become destructive in a laboratory context. A well‐known case is the automatic conversion of gene symbols into dates. In a survey of supplementary files from genomics journals, Ziemann and colleagues reported that a substantial fraction of published gene lists had been corrupted when spreadsheet software converted symbols such as SEPT9 into date formats such as “9‐Sep” [8, 9, 10]. The problem became so persistent that the Human Gene Nomenclature Committee ultimately renamed many affected genes to avoid further corruption [11]. This example highlights a risk inherent to automated, heuristic‐driven data interpretation by spreadsheet software.

A second failure mode involves long numeric identifiers. Laboratory accession numbers and specimen barcodes are often lengthy strings that function as identifiers rather than quantities. When imported into a spreadsheet, these values may be coerced into scientific notation or rounded due to numeric precision limits, altering the identifier and breaking the link between analytical results and the underlying specimen. A related issue arises with identifiers that contain leading zeros, such as medical record numbers. Automatic numeric typing removes these zeros, which complicates referencing patient charts and can cause failures in merging with laboratory information system reports.

### 2.3 Case Study in Spreadsheet Failure

These concerns are far from theoretical. In the mid‐2000s, researchers at Duke University reported genomic signatures purported to predict which chemotherapeutic agents would be most effective for individual patients' tumors. Their findings were published in high‐impact journals, and prospective clinical trials were initiated to test whether the signatures could improve patient outcomes. When independent statisticians Keith Baggerly and Kevin Coombes attempted to reproduce the analyses, they found that the underlying analytical workflow could not be determined from the published materials. By doggedly attempting to reconstruct the analytical methods using only raw data and published outputs, a process they termed “forensic bioinformatics,” they identified multiple data management errors, including a likely row‐shift error within a spreadsheet that misaligned gene probe identifiers with expression values and inverted labels for treatment‐sensitive and treatment‐resistant patients [12]. The trials were ultimately halted, multiple papers were retracted, and the careers of the investigators were upended [13].

The spreadsheets used by the researchers permitted, and in some cases encouraged, these data management errors. More importantly, reliance on a manual, graphical user interface‐based workflow meant there was no transparent, reviewable record of the analytical steps taken. The sequences of clicking, dragging, and copying leave no durable trace once the file is saved. As demonstrated by the more than 1500 h of forensic analysis required to reconstruct the workflow, the absence of an explicit, ordered record rendered these errors extraordinarily difficult to identify during peer review [14].

This phenomenon is not unique to biomedical research. Documented failures related to spreadsheet software have occurred in various high‐stakes sectors. Notable examples include significant trading losses at JPMorgan Chase during the London Whale incident [15], the omission of over 16 000 COVID‐19 cases in the United Kingdom [16], and a spreadsheet error that overstated the link between national debt and economic growth, thereby affecting critical public policy discussions [17].

Analytical errors are a persistent problem in data analysis workflows [18]. A fundamental limitation of spreadsheets is that they hinder detection of these errors. Without an explicit record of how results were generated, review and auditing become difficult, increasing the likelihood that errors persist undetected. Given that analytical conclusions in laboratory medicine directly inform clinical decisions, workflows that cannot be reconstructed, reviewed, and audited fall short of what should be expected in both clinical laboratory practice and biomedical research. A central requirement for addressing these shortcomings is reproducibility [19].

Spreadsheet software remains widely used for data analysis. This may reflect two intuitive but problematic lines of reasoning. The first is a belief that the effort required to learn a new analytical tool outweighs the risk of continuing with familiar software. The second is a belief that extensive experience with spreadsheets and awareness of their known quirks is sufficient to manage or avoid error. In practice, these assumptions underestimate the risks of spreadsheet‐based data analysis, which have repeatedly manifested as large‐scale and, in some cases, catastrophic data failures [5].

### 2.4 Reproducibility

Reproducibility refers to the ability of an independent investigator to obtain the same results using the same raw data and the same analytical procedures. Concerns about reproducibility have intensified across many scientific disciplines, as multiple investigations have shown that published findings often cannot be reproduced. Within clinical laboratories, data analysis is fundamental, and reproducibility should be considered an expectation. ISO 15189:2022 requires laboratories to conduct a wide range of analyses, including method validation and continuous quality monitoring, throughout all phases of testing. These analyses must be documented in a way that enables review and evaluation, ensuring the appropriate use of statistical methods [20, 21]. R offers laboratories a transparent and reproducible platform to perform these essential analytical tasks, supporting both regulatory compliance and operational excellence.

### 2.5 Script‐Based Analysis

**FIGURE 2:**
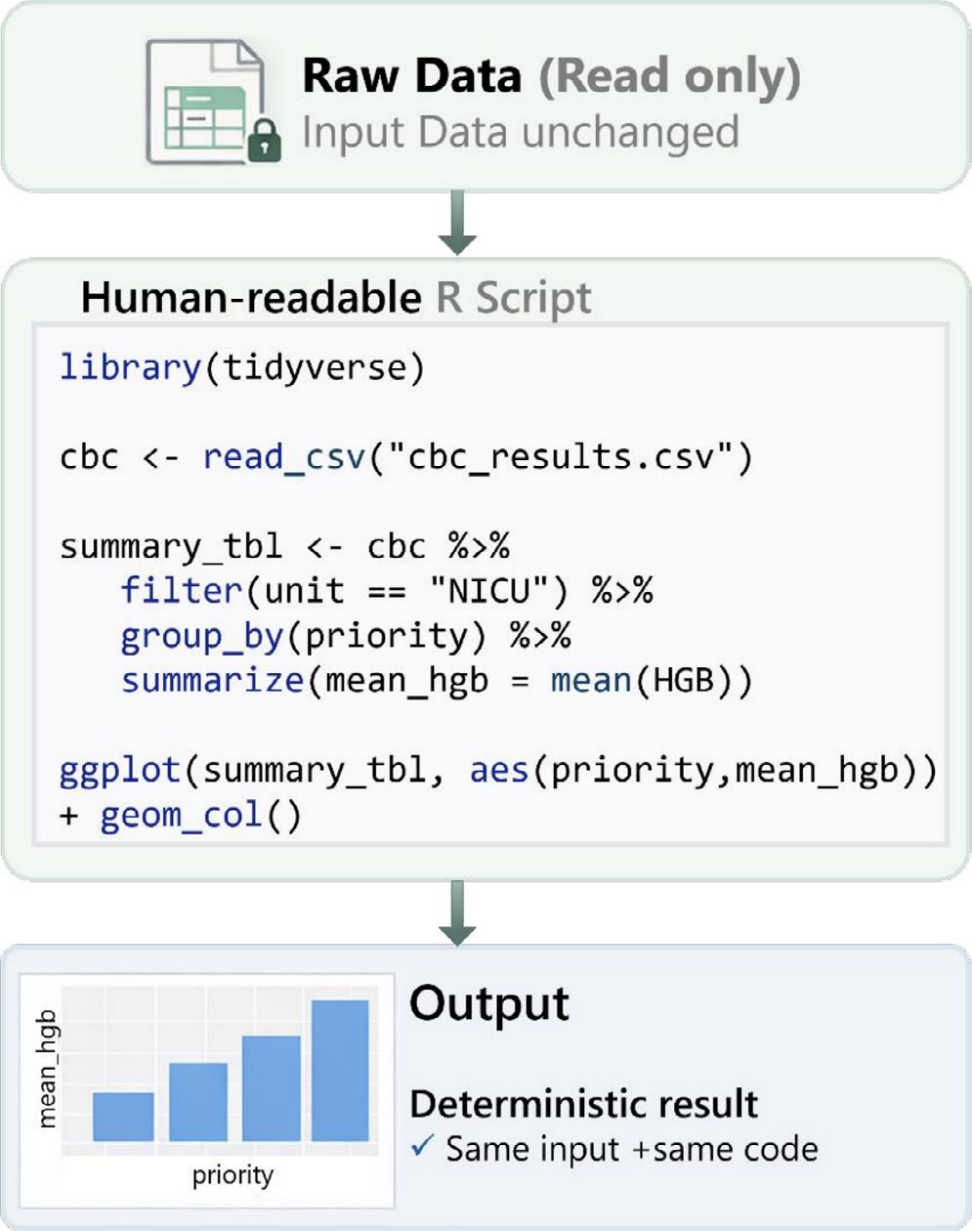
Example of a simple script‐based analysis in R. Raw data are treated as read‐only inputs and transformed through an explicit, ordered sequence of operations recorded in a human‐readable R script. The same input data and code can be rerun at any time to deterministically regenerate the same tabular and graphical outputs.

Script‐based data analysis provides an alternative to traditional spreadsheet workflows. Instead of relying on manual manipulation, script‐based workflows such as those used in R encode an analysis as a series of steps in which each is represented as executable code, establishing a permanent ordered record of data transformations and calculations (Figure 2). In these workflows, raw data are treated essentially as read‐only inputs, with all manipulations applied to derived objects rather than altering the original dataset, thereby preserving data integrity. This approach guarantees clarity and facilitates comprehension of the analysis for any reader, both in present and future reviews. Moreover, this architecture provides the ability to rerun the script with new or updated data at any time, making the analytical process more efficient and ensuring consistency [22].

### 2.6 R for Data Analysis

Among the available tools for script‐based data analysis R [23] represents a widely adopted option, particularly within biomedical research and laboratory medicine. R was developed in the early 1990s at the University of Auckland by Ross Ihaka and Robert Gentleman as an open‐source language for statistical computing and data visualization, influenced by the S language developed at Bell Labs [24]. From its inception, R was designed to operate naturally on tabular data and to support advanced statistical modeling and graphical techniques. Today, R serves as a cornerstone for data science and bioinformatics.

R is highly extensible due to its robust ecosystem of user‐contributed add‐on packages that extend its capabilities. This system of packages allows users to add specialized functions for tasks such as data manipulation, visualization, machine learning, bioinformatics, and more, making R adaptable for a wide range of scientific and analytical applications. Its open‐source model ensures free access, making it particularly suitable for environments with limited financial resources that cannot afford proprietary software licenses. Together, these capabilities position R as a practical and scalable foundation for reproducible data analysis in modern laboratory practice.

## 3 Practical Applications for Laboratory Hematology

The value of script‐based data analysis lies not only in reproducibility but in its virtually unlimited analytical capability. Modern laboratory practice increasingly demands statistical methods that exceed what spreadsheet software can easily support. While spreadsheets may suffice for basic descriptive analyses, they quickly reach their limits as analytical complexity increases. Some CLSI protocols require sophisticated approaches that are difficult to implement, validate, or maintain in traditional spreadsheet environments. R provides laboratories with the statistical capabilities needed for these types of analyses (Table 1).

**TABLE 1:** Sample R packages relevant to lab hematology.

Domain	R package	Primary function
Method comparison	mcr	Method comparison using Deming and Passing–Bablok regression
Diagnostic performance	pROC	ROC curve analysis and AUC estimation
Measurement uncertainty	metRology	Uncertainty estimation, calibration, and propagation
Reference intervals	referenceIntervals	Parametric and non‐parametric RI estimation
Indirect reference intervals	refineR, TMC, kosmic, reflimR	Modeling healthy populations from routine patient data
Age‐dependent Reference interval modeling	gamlss, quantregGrowth	Modeling smooth, age‐continuous centile curves
Quality control	qcc	Statistical process control and control charting
Flow cytometry	flowCore	Import and transformation of FCS data
Data wrangling	dplyr	Reproducible data manipulation and transformation
Visualization	ggplot2	Publication quality statistical graphics
Reporting	rmarkdown/quarto	Reproducible reports and dashboards
Reporting/dashboards	flexdashboard	Interactive dashboards for QC, KPIs, and operational monitoring

### 3.1 Method Comparison

When comparing two clinical measurement methods, ordinary least squares regression is typically inappropriate because it assumes the x‐axis variable is measured without error. CLSI EP09 recommends regression approaches that account for imprecision in both methods [25]. The R package **mcr** implements these models directly, including Deming and Passing–Bablok regression, enabling laboratories to quantify method relationships, estimate bias at clinical decision points, and generate confidence intervals in a manner aligned with CLSI guidance [26].

In addition to regression‐based method comparison, Receiver Operating Characteristic (ROC) curve analysis is indispensable for evaluating the diagnostic accuracy of laboratory tests. The **pROC** package provides comprehensive tools for generating ROC curves, calculating the area under the curve (AUC), and visualization and confidence interval estimation [27].

### 3.2 Measurement Uncertainty, Calibration, and Uncertainty Propagation

The **metRology** package in R offers specialized tools for assessing measurement uncertainty, performing calibration, and propagating uncertainties [28]. Such calculations have become even more critical in laboratory hematology given updated requirements of ISO 15189. The package implements advanced algorithms for uncertainty evaluation, including Monte Carlo methods, and supports both linear and non‐linear calibration models. Laboratories can use metRology to generate uncertainty budgets, combine uncertainties from multiple sources, and document traceability as required by accreditation standards.

### 3.3 Reference Intervals

Establishing appropriate reference intervals (RIs) is a core requirement in laboratory hematology. R provides laboratories with validated statistical frameworks for RI estimation that extend well beyond basic percentile calculations. The R package **referenceIntervals** supports both parametric and non‐parametric RI estimation, enabling laboratories to apply transformations or distribution‐based methods [29]. Indirect RI estimation approaches leverage patient data generated during routine practice rather than healthy volunteers. Indirect methods, implemented in packages such as **refineR** [30], **TMC** [31], **kosmic** [32], and **reflimR** [33] allow laboratories to model the underlying healthy population within mixed clinical datasets, making RI development feasible in settings where direct recruitment is impractical, such as pediatric or geriatric populations. A user‐friendly web application is available as an alternative to R execution for those not yet proficient in R programming [34]. Beyond the estimation of static intervals, age‐dependent reference interval modeling is indispensable in particular for pediatric laboratory medicine, where analyte distributions shift dynamically across developmental stages. R provides robust support for this through packages such as **gamlss** [35] and **quantregGrowth** [36], which facilitate the generation of smooth, age‐continuous centile curves. To facilitate clinical translation, user‐facing tools such as the **AdRI** [37] web application provide an accessible interface for applying these complex modeling frameworks in practice.

### 3.4 R in Quality Control

Traditional laboratory QC workflows typically rely on visual or semi‐automated inspection of Levey‐Jennings charts. Packages such as **qcc** provide a reproducible framework for implementing control charts and process monitoring that can be tuned to laboratory‐specific criteria [38]. By encoding QC logic in scripts rather than manual chart review, laboratories can standardize QC assessment, document decision rules explicitly, and apply identical criteria across large health networks, enabling systematic detection of both random and systematic variation over time.

### 3.5 Use of R in Flow Cytometry Laboratories

Flow cytometry laboratories generate large, high‐dimensional datasets that often exceed the analytical scope of traditional manual review approaches. Script‐based analysis using packages such as **flowCore** supports scalable, repeatable pipelines for these data [39]. Script‐based analysis is particularly valuable in this context because it supports scalable, repeatable pipelines across large flow cytometry datasets. When analyses involve many FCS files, scripting allows the same transformations, gating logic, and quality‐control checks to be applied consistently across cohorts. Pipelines can be re‐run after parameter adjustments, and standardized outputs can be regenerated automatically while preserving a record of the analytical process. Although R is not typically used for primary diagnostic gating in routine clinical flow laboratories, it has become an important analytical platform in research, core facility, and translational settings.

### 3.6 Machine Learning, AI, and Digital Imaging

While the examples above focus on routine statistical and operational tasks, the same script‐based foundations enable more advanced analytical capabilities. R provides a comprehensive toolset for classical machine learning and emerging AI‐based techniques that extend laboratory hematology beyond descriptive analysis into prediction, pattern recognition, and decision support. Within the R ecosystem, the **tidymodels** [40] framework provides a consistent, cohesive structure for the entire modeling lifecycle. Furthermore, when a workflow requires specialized Python libraries, the **reticulate** [41] package allows R to execute Python code within a single reproducible pipeline. This effectively provides laboratory professionals with seamless access to the broader AI landscape, including industry‐standard tools such as **scikit‐learn**, **TensorFlow**, and **PyTorch**. Using the same reproducible scripting principles, laboratories can develop, validate, and audit models for tasks such as risk stratification, sample flagging, and abnormal population detection.

Beyond tabular data, R increasingly supports quantitative image analysis and digital pathology workflows through packages that enable feature extraction, dimensionality reduction, clustering, and model‐based classification. Although these advanced applications are not yet routine, they illustrate how adopting R for everyday analytical needs simultaneously positions laboratories to engage with more advanced techniques as these approaches continue to mature and enter clinical practice.

### 3.7 Applications of R in Laboratory Medicine

The application of R in the clinical laboratory is well documented in the academic literature, spanning routine analytical and operational tasks as well as more advanced applications. While a comprehensive survey is beyond the scope of this publication, selected representative examples illustrate these capabilities. Bahar et al. used the mcr package described above to create an R‐based web application implementing Deming and Passing‐Bablok regression as well as a variety of Bland–Altman plots, enabling generation of downloadable analysis reports [42]. Automated quality‐control review using R was reported by Stickle and colleagues, who reduced monthly QC review time from more than 12 h to less than 4 h [43]. R is also capable of automating laboratory interfaces, allowing data to be transferred automatically instead of manually. In one example, Mobini and colleagues described an R‐based data pipeline to support COVID‐19 testing that parsed instrument outputs, generated worklists and plate‐map review tools, and transferred data into the LIS, enabling pooled PCR analysis while replacing extensive manual transcription [44]. R has been extensively used for lab KPI monitoring. Maury et al. reported the use of R to create dashboards that continuously extract COVID‐19 testing data from the LIS and display real‐time volumes, turnaround times, and operational bottlenecks to support decision‐making [45]. In summary, adopting R supports reproducible data analysis while simultaneously improving laboratory efficiency and providing a foundation for advanced techniques, including machine learning, that are increasingly relevant to modern laboratory practice.

### 3.8 Data Integration and Automated Reporting

The transition to script‐based analysis further enables the full automation of the laboratory data lifecycle, from ingestion to dissemination. While spreadsheets often rely on manual data entry, “copy‐paste”, or data export workflows, R can connect directly to a variety of data sources, including databases and web‐based APIs. Laboratory professionals can create scripts to directly access the LIS or middleware. This ensures the analyzed data is always accurate and comes from the original “source of truth”. Combined with the ability to schedule scripts (e.g., via cron jobs or similar task schedulers), recurring tasks can be automated such as QC data analysis, key performance indicator (KPI) monitoring, and productivity reporting.

Once the data is processed, R supports a diverse array of reproducible output formats as well. Frameworks such as **Quarto** and **RMarkdown** can automatically render results into HTML, PDF, PowerPoint, Excel or Word (using **officer**). By replacing manual data aggregation and cleansing with an automated pipeline, these tools significantly reduce the risk of human error while liberating laboratory staff for more complex diagnostic tasks. **First Steps with R**.

Pathologists and laboratory professionals can begin learning R by utilizing a range of informal learning resources. Online learning platforms such as edX and Coursera host numerous courses in R, some of which are directed specifically at trainees and professionals in the health sciences. DataCamp, a commercial site for data science learning, provides interactive R courses, with some content available for free. Posit (formerly known as RStudio) is a company that develops open‐source and commercial software tools to support data science workflows, with a primary focus on R. Posit provides educational resources and curated learning paths that teach R for a variety of use cases. In addition, professional organizations such as the Association for Pathology Informatics (API) and the Association for Diagnostics & Laboratory Medicine (ADLM) have hosted formal R workshops specifically for pathologists and laboratory professionals. Academic institutions also provide periodic R‐based training focused on health data science and research reproducibility. Essential texts such as *Hands‐On Programming with R* and *R for Data Science* are available online and are useful references as well [46, 47]. For the motivated laboratory professional, a wealth of resources and training opportunities are available to support training in R.

### 3.9 R and Large Language Models

The advent of large language models (LLMs) has lowered barriers to working with R while preserving the transparency and auditability of code‐based analysis. LLMs can support learning by generating example scripts, explaining syntax and errors, and translating users' conceptual descriptions of an analysis into executable R code. Beyond instructional use, LLMs can be integrated directly into R‐based workflows, enabling R scripts and applications to call LLMs directly for tasks such as data summarization, feature annotation, and user interaction.

### 3.10 Getting Started With R

R is freely available through the Comprehensive R Archive Network (CRAN), which provides standardized distributions for all major operating systems. Installation is straightforward, with a variety of options to deploy R in local, server‐based, or institutional computing environments.

In practice, R is most often used within an integrated development environment (IDE), such as RStudio [48], which provides a structured workspace for project organization, script development, and data visualization. For collaborative or regulated environments, server‐based deployments allow centralized access while maintaining consistent analytical environments across users. Version control is a natural complement to script‐based analysis and is natively supported within modern R IDEs. By managing R projects under systems such as Git, laboratories can track changes over time, review prior versions, and support collaborative development.

## 4 Conclusion

The convergence of increasingly complex laboratory datasets and the rapid maturation of artificial intelligence makes this an opportune moment for laboratory hematology to accelerate adoption of modern, script‐based analytical tools such as R. Reproducible environments like R represent one practical element of data literacy, defined here as the ability to critically acquire, manage, analyze, interpret, and communicate data. Proficiency in R and related tools enables laboratory professionals to move beyond passive data handling toward transparent, auditable analysis. As these competencies gradually become more common within the field, laboratories will be better positioned to extract biological insight from routinely generated data, to evaluate emerging AI methods with appropriate rigor, and to translate quantitative findings into improved diagnostics and patient care. In this sense, investment in expertise with tools such as R, alongside broader development of data literacy, supports the continued evolution of laboratory hematology as a data‐driven field.

## Funding

The author has nothing to report.

## Data Availability

Data sharing not applicable to this article as no datasets were generated or analysed during the current study.
